# Retracted: Qingyihuaji Formula Inhibits Pancreatic Cancer and Prolongs Survival by Downregulating Hes-1 and Hey-1

**DOI:** 10.1155/2021/8230693

**Published:** 2021-02-20

**Authors:** Evidence-Based Complementary and Alternative Medicine

*Evidence-Based Complementary and Alternative Medicine* has retracted the article titled “Qingyihuaji Formula Inhibits Pancreatic Cancer and Prolongs Survival by Downregulating Hes-1 and Hey-1” [1], due to concerns identified with image overlap in Figure 6 as raised on PubPeer [2]. Figure 6(a) appears to have areas of overlap between the control and Gem panels. Figure 6(b) appears to have areas of overlap between the QYHJ and Gem panels.

The authors were not able to provide a satisfactory response to these concerns and it is therefore being retracted with the agreement of the authors and the editorial board.
